# *Cryptorchestia ruffoi* sp. n. from the island of Rhodes (Greece), revealed by morphological and phylogenetic analysis (Crustacea, Amphipoda, Talitridae)

**DOI:** 10.3897/zookeys.652.11252

**Published:** 2017-02-06

**Authors:** Domenico Davolos, Elvira De Matthaeis, Leonardo Latella, Ronald Vonk

**Affiliations:** 1 INAIL, Research, Certification, Verification Area, Department of Technological Innovations and Safety of Plants, Products and Anthropic Settlements (DIT), Rome, Italy; 2 Department of Biology and Biotechnology 'Charles Darwin', Sapienza University of Rome, Viale dell'Università, 32, - 00185 Rome, Italy; 3 Museo Civico di Storia Naturale of Verona, Lungadige Porta Vittoria 9 – 37129, Verona, Italy; 4 Naturalis Biodiversity Center, P.O. Box 9517, 2300 RA Leiden, The Netherlands; 5 Institute for Biodiversity and Ecosystem Dynamics, University of Amsterdam, Science Park 904, Amsterdam 1098 XH, The Netherlands

**Keywords:** biogeography, freshwater, Greek islands, molecular phylogeny, taxonomy

## Abstract

A new *Cryptorchestia* species, *Cryptorchestia ruffoi* Latella & Vonk, **sp. n.** from the island of Rhodes in south-eastern Greece, can be distinguished on the basis of morphological and phylogenetic data. Morphological analysis and DNA sequencing of mitochondrial and nuclear protein-coding genes indicated that this species is related to *Cryptorchestia cavimana* (Cyprus) and *Cryptorchestia garbinii* (Mediterranean regions, with a recent northward expansion). Results supported a genetic separation between the *Cryptorchestia* species of the east Mediterranean regions and those of the northeast Atlantic volcanic islands examined in this study (*Cryptorchestia canariensis*, *Cryptorchestia gomeri*, *Cryptorchestia guancha*, and *Cryptorchestia stocki* from the Canary islands, *Cryptorchestia monticola* from Madeira, and *Cryptorchestia chevreuxi* from the Azores). The Mediterranean and Atlantic *Cryptorchestia* species appear to be also morphologically distinct. *Cryptorchestia ruffoi* **sp. n.**, *Cryptorchestia cavimana*, *Cryptorchestia garbinii*, and *Cryptorchestia kosswigi* (Turkish coast) clearly have a small lobe on the male gnathopod 1 merus. This character was the main diagnostic difference between *Cryptorchestia* (*sensu* Lowry, 2013) and *Orchestia*. However, among the six northeast Atlantic island *Cryptorchestia* species only *Cryptorchestia stocki* has a small lobe on the merus of gnathopod 1. Reduction or loss of the lobe in the Atlantic Island species cannot be ruled out; however, molecular phylogenetic analysis leads us to presume that this lobe independently evolved between the east Mediterranean *Cryptorchestia* species and *Cryptorchestia stocki* from Gran Canaria.

## Introduction

The genus *Cryptorchestia* Lowry & Fanini, 2013 is partitioned from *Orchestia* Leach, 1814, and is associated with freshwater-soaked leaf litter (Lowry and Fanini 2013). *Cryptorchestia* species from the East Mediterranean region were found in riparian habitats (Ruffo et al. 2014; present study), while the species from the Canary Islands, Madeira, and Azores (North East Atlantic area) live in humid, evergreen broadleaf laurel forest (laurisilva) (Stock 1989; Stock and Boxshall 1989; Ruffo 1990; Stock and Abreu 1992; Villacorta et al. 2008).

**Figure 1.**
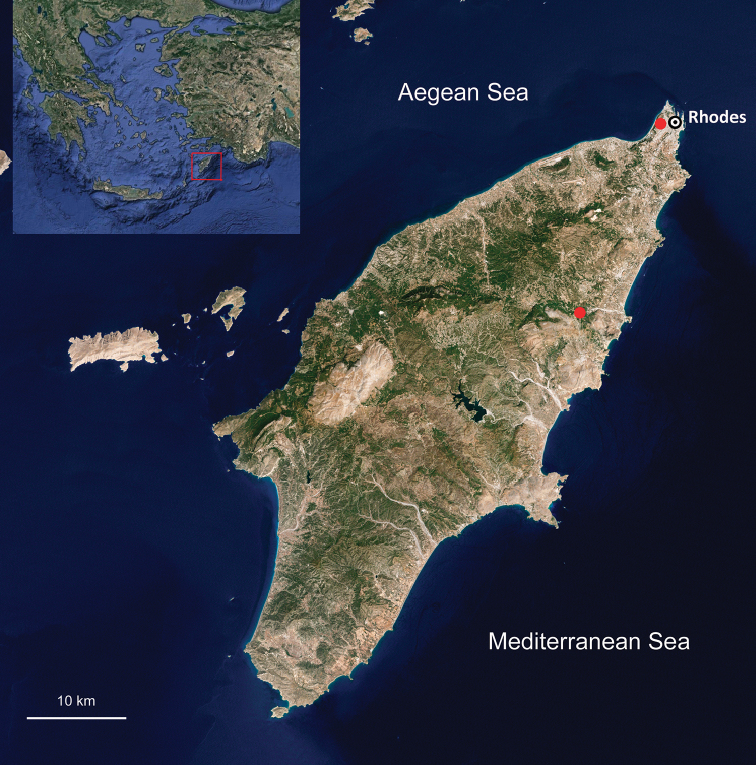
Occurrence of *Cryptorchestia ruffoi* sp. n. on Rhodes, Greece (MSNVR). In the north, near the town of Rhodes, the locality of 1928 on Monte Smith. The other place represents small streams in the vicinity of Psintos, Epta Piges spring and stream.

Here *Cryptorchestia ruffoi* sp. n. is described from the island of Rhodes, Greece. Specimens were first collected during the military occupation of Rhodes by the Italian army in 1928: the geologist Angelo Pasa of the Museo Civico di Storia Naturale of Verona found two talitrid specimens in a spring on Monte Smith (Fig. 1). Sandro Ruffo subsequently identified the specimens as belonging to a probable new taxon with morphological resemblances to *Cryptorchestia cavimana* (Heller 1865) and stored them in the Amphipoda collection of the Verona Museum. In 2010, one of us (L.L.) organised an expedition to Rhodes to find more talitrid amphipods from freshwater springs and succeeded in collecting more than 30 specimens.

A morphological characterisation forms part of this study. In order to gain insight into its position in relation to other *Cryptorchestia* species of the Mediterranean and North East Atlantic areas, a phylogenetic analysis was performed on DNA sequences of both mitochondrial (mt) and nuclear gene fragments, cytochrome oxidase I (COI), and histone H3 (H3), respectively. In particular, focus was on *Cryptorchestia ruffoi* sp. n. from Rhodes, *Cryptorchestia cavimana* (Heller, 1865) from Cyprus, *Cryptorchestia garbinii* Ruffo, Tarocco and Latella, 2014, from mainland Europe, *Cryptorchestia canariensis* (Dahl, 1950), *Cryptorchestia gomeri* (Stock, 1989), *Cryptorchestia guancha* (Stock & Boxshall, 1989), and *Cryptorchestia stocki* (Ruffo, 1990) from the Canary Islands, *Cryptorchestia monticola* (Stock & Abreu, 1992) from Madeira, and *Cryptorchestia chevreuxi* (De Guerne, 1887) from Terceira, Azores.

## Material and methods

**Figure 2.**
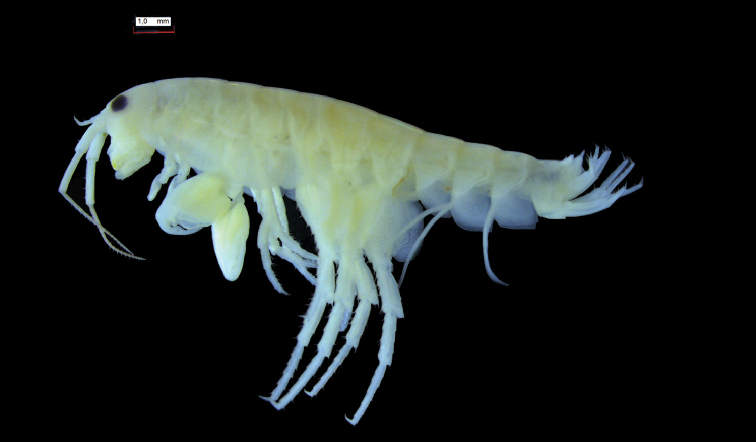
*Cryptorchestia ruffoi* sp. n., paratype, male (MSNVR). Lateral view.

**Morphology.** The specimens analysed were collected in two localities from Rhodes (Fig. 1) and stem from different years with a gap of 82 years between them. A total of 37 specimens was examined for the description and measurements of the new species. Thirty one specimens were preserved in 70% ethanol, two mounted in glass slides in Faure's medium, two mounted on stubs for scanning electronic microscope (SEM) photography, and two used for molecular analyses. SEM photographs were obtained with a Zeiss EVO 40 XVP Scanning Electronic Microscope at the MUSE-Science Museum of Trento. The photo of a male paratype (Fig. 2) was obtained with a stereo microscope Leica M 165c, mounted with a Leica DFC450 camera at the Museo Civico di Storia Naturale of Verona. Type material is deposited in the Museo Civico di Storia Naturale of Verona (MSNVR), Verona, Italy, and the Naturalis Biodiversity Center (RMNH), Leiden, The Netherlands.

**PCR amplification and DNA sequencing.** Specimens of *Cryptorchestia ruffoi* sp. n. from Rhodes, *Cryptorchestia cavimana* from Cyprus, *Cryptorchestia garbinii* from Europe and Macedonia, *Cryptorchestia canariensis*, *Cryptorchestia gomeri*, *Cryptorchestia guancha*, and *Cryptorchestia stocki* from the Canary Islands, *Cryptorchestia monticola* from Madeira, and *Cryptorchestia chevreuxi* from Terceira, Azores were stored in ethanol (Table 1). *Platorchestia platensis* from Capri island, Italy, was included in this study as the outgroup species (Table 1).

**Table 1.** Mediterranean and North-East Atlantic *Cryptorchestia* species employed in the molecular analysis. Shown are the mitochondrial COI gene region (363 bp), the H3 histone gene fragment (330 bp), the sampling locations, and the GenBank accession number (acc. no.). *Platorchestia platensis*, used in this study as outgroup species, is also reported. NA = not available.

Species	Sampling locality	acc. no.		
		COI	H3	Reference
*Cryptorchestia canariensis* (Dahl, 1950)	Gran Canaria, Canary Islands, Spain	KY225807	KY225817	present study
*Cryptorchestia cavimana* (Heller, 1865)	Troodos Mountains, Cyprus	KY225808	KY225818	present study
*Cryptorchestia chevreuxi* (de Guerne, 1887) (ZMA.CRUS.A.108.587; Leiden Museum)	Terceira, Azores, Portugal	NA	KY225819	present study
*Cryptorchestia garbinii* Ruffo, Tarocco & Latella 2014	Lake Ohrid, Macedonia	KY225809	KY225820	present study
	Dijon, France	KY225810	KY225821	present study
	Latium, Italy	KY225811	KY225822	present study
*Cryptorchestia guancha* (Stock & Boxshall, 1989)	Zapata, Tenerife, Canary Islands, Spain	KY225812	KY225823	present study
*Cryptorchestia gomeri* (Stock, 1989)	La Gomera, Canary Islands, Spain	NA	AM748658	Villacorta et al. 2008
*Cryptorchestia monticola* (Stock & Abreu 1992) (paratype; Leiden Museum)	Madeira Island, Portugal	KY225813	KY225824	present study
*Cryptorchestia ruffoi* Latella & Vonk, sp. n.	Rhodes Island, Greece	KY225814	KY225825	present study
*Cryptorchestia stocki* (Ruffo, 1990) (paratype; Museo Civico di Storia Naturale, Verona)	Gran Canaria, Canary Islands, Spain	KY225815	KY225826	present study
*Platorchestia platensis* (Krøyer, 1845)	Capri Island, Italy	KY225816	KY225827	present study

Genomic DNA was extracted from pereopods or whole organisms using QIAamp DNA Mini kit (QIAGEN). A PCR product of ca. 400 base pairs (bp) was amplified from the gene encoding the mt COI (some of our samples were old museum specimens in which mitochondrial DNA was degraded and consequently produced only short DNA sequences). The PCR-mediated reaction was performed using the primers BI-COI and SUBIR cited in Davolos and Maclean (2005). The PCR amplification conditions were 2 min at 95 °C, followed by 35 cycles, each consisting of 10–15s at 95 °C, 15s at 48–50 °C and 10s at 72 °C; the final PCR extension step lasted 10 min at 72 °C. The amplified fragments were checked by electrophoresis in 1% agarose gels and then used as templates for cycle sequencing reactions (BigDye chemistry) followed by DNA sequencing (ABI Prism 3130 capillary sequencer) using BI-COI and SUBIR primers. In addition, a fragment of ca. 350 bp of the gene encoding the nuclear histone H3, was PCR amplified using the primers H3Of and H3Or cited in Davolos and Pietrangeli (2014). The PCR cycling parameters were 2 min at 95 °C, followed by 35 cycles, each consisting of 10s at 95 °C, 10s at 48–52 °C and 5s at 72 °C; the final extension step lasted 10 min at 72 °C. The PCR products were verified and then sequenced using H3Of and H3Or primers, as above described.

**Bioinformatic analysis.** The nucleotides obtained in this study and the amino acid residues inferred were compared with sequence data accessible in the GenBank databases at the National Center for Biotechnology Information (NCBI; http://www.ncbi.nlm.nih.gov) using the BLASTN algorithm. The nucleotide sequence alignments were made in ClustalX (1.8) using the default parameters. Evolutionary analyses for the combined mt COI and nuclear histone H3 gene sequences were conducted in MEGA7 (Kumar et al. 2016). The evolutionary history was inferred by using the Maximum Likelihood method based on the General Time Reversible model (Nei and Kumar 2000). The tree with the highest log likelihood (-2646.1008) was used. Initial trees for the heuristic search were obtained automatically by applying Neighbor-Join and BioNJ algorithms to a matrix of pairwise distances estimated using the Maximum Composite Likelihood (MCL) approach, and then selecting the topology with superior log likelihood value (Fig. 10). A discrete Gamma distribution was used to model evolutionary rate differences among sites (5 categories (+G, parameter = 0.1808)). For Maximum Likelihood analysis, bootstrap resampling was performed with 1000 replications. The novel annotated sequences from the COI and the histone H3 genes from *Cryptorchestia* species of this study have been submitted to the GenBank (NCBI) database (Table 1).

## Systematics

### Order Amphipoda Latreille, 1816 Suborder Senticaudata Lowry & Myers, 2013 Family Talitridae Rafinesque, 1815 Genus *Cryptorchestia* Lowry & Fanini, 2013

#### Cryptorchestia ruffoi

Taxon classificationAnimaliaAmphipodaTalitridae

Latella & Vonk sp. n.

http://zoobank.org/1F792FCC-9F42-48A4-A445-2814E0B1F8FF

Figs 2 , 3 , 4 , 5 , 6 , 7 , 8 , 9

##### Type locality.

Island of Rhodes, Greece. A spring on Monte Smith and in streams flowing out of the springs called Epta Pyges (Seven Springs), Municipality of Archangelos.

##### Etymology.

Species named after Sandro Ruffo (1915 – 2010), tutor to us all, who worked on Mediterranean talitrid amphipods from an early stage.

**Type specimens.** Holotype male (15.7 mm): Greece, Rhodes Island, Rhodes, Monte Smith, near a little spring, June 1928, (labelled: Rodi VI-928 Monte Smiti, vicino piccola sorgente) (MSNVRCr 589). Paratypes: 9 males, 25 females; Greece, Rhodes, Psintos, Epta Piges spring's stream, 36°15'10.9'N - 28°06'49.3'E, 7/8-VII-2010, A. & L. Latella, V. Lencioni leg.: 1 male (vial RMNH.CRUS.A.5070 + slides RMNH.CRUS.21512–21515), 1 female (slides RMNH.CRUS.21516–21518), 2 male, 5 female RMNH.CRUS.A.5071; 4 males, 17 females MSNVRCr 590–611)

##### Additional material examined.

*Cryptorchestia garbinii* Ruffo, Tarocco & Latella, 2014 : Italy, Lombardy, Brescia province, Lake Garda, between Desenzano and Padenghe, 45°29'N–10°30'E, V-1895, A. Garbini leg.; Venetia, Verona province, Lake Garda, Peschiera, 45°26'51"N–10°41'39"E, 18-IV-2010 L. Latella, V. Lencioni leg.

*Cryptorchestia cavimana* (Heller, 1865): Cyprus, Troodos Mountains, Kaledonia falls, 1250 m a.s.l., 9-VI-2000 M. Tarocco leg.; Cyprus, Troodos Mountains, between Prodromos and Troditissa, 1300 m a.s.l., 10-VI-2000, M. Tarocco leg.

##### Diagnosis.

Gnathopd 2 propodus sinusoid palmar margin with its strongest incursion close to the anterior side. Maxilla 1 with vestigial palp present on the outer lobe. Pereopod 5 with four groups of setae on the posterior margin in females and five groups in the males. Antenna first flagellum segment sometimes fused with second, forming a longer unit.

##### Description.

**Figure 3.**
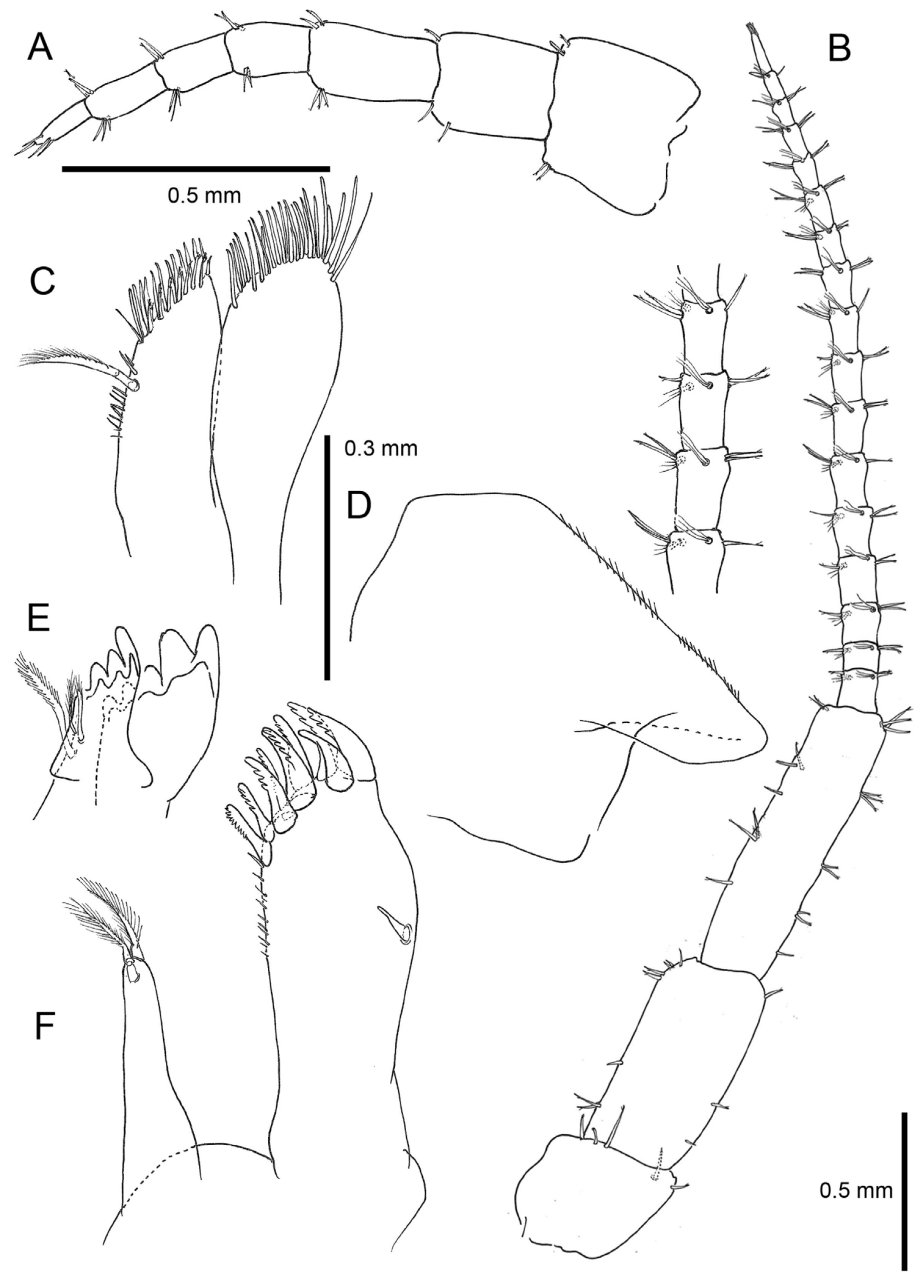
*Cryptorchestia ruffoi* sp. n., paratype male, 10.4 mm (RMNH) **A** antenna 1 **B** antenna 2 **C** maxilla 2 **D** lower lip **E** lacinia mobilis, left mandible **F** maxilla 1.

**Figure 4.**
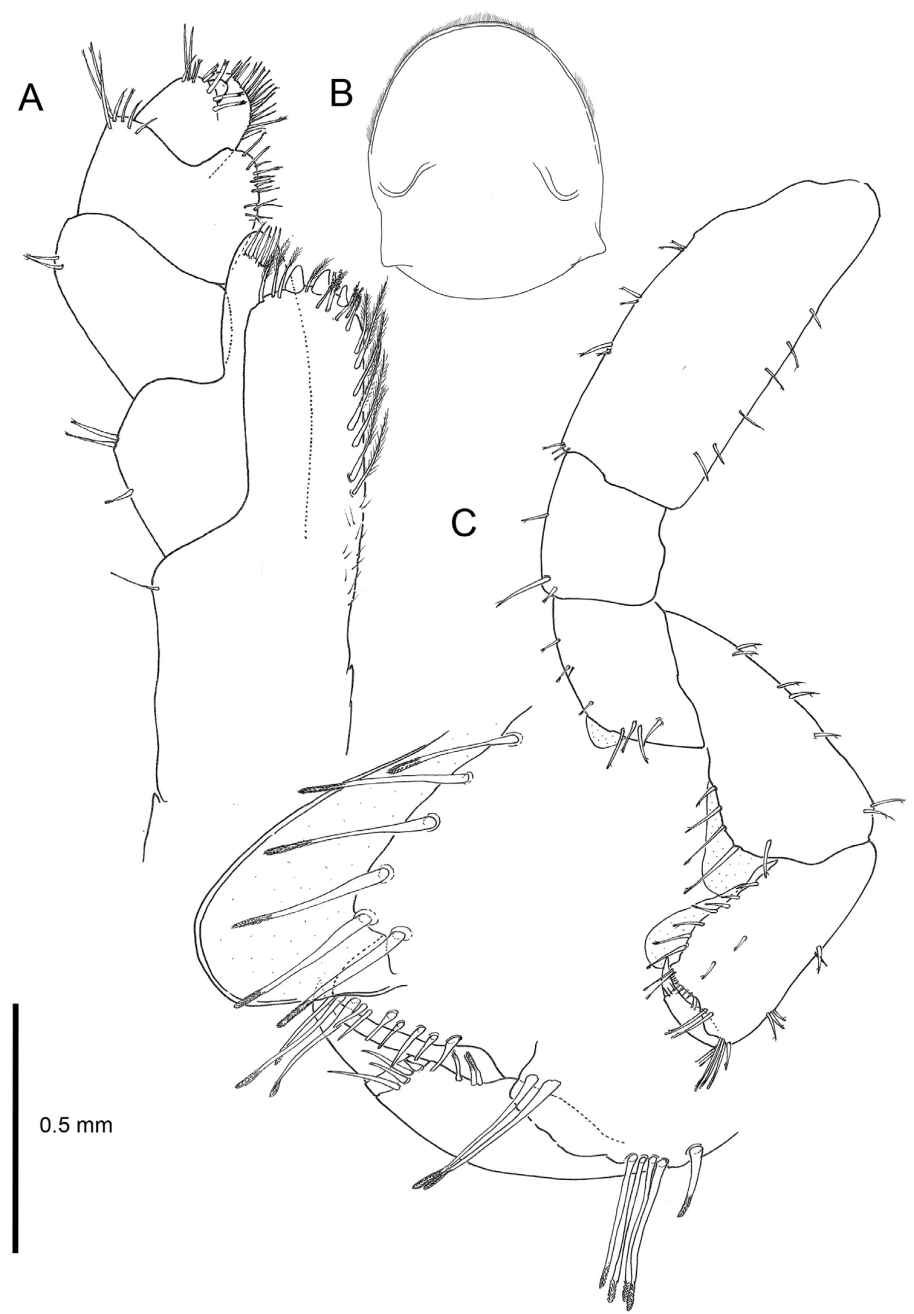
*Cryptorchestia ruffoi* sp. n., paratype male, 10.4 mm (RMNH) **A** maxilliped **B** upper lip **C** gnathopod 1.

Based on adult males with an average length of 12.20 mm (Table 2). **Head**. *Eyes* large, subcircular, black. *Antenna 1* (Figs 2, 3A) short, 1.46 mm long in average, slightly longer than peduncle article 4 of antenna 2, peduncle segment 2 slightly shorter than segment 3, flagellum with four articles. *Antenna 2* (Figs 2, 3B) long, little shorter than half of the body length, article 5 longer than article 4, flagellar articles with four tufts of setae of which two spring from a hollow in a series of three fine setules, flagellum relatively short with 16–19 articles. In some antennae the first flagellum segment is fused with the second and forms a longer unit. *Labrum* (Fig. 4B) and *labium* (Fig. 3D) with very fine setules on anterior margin. *Mandible* (Fig. 3E) left with 4-dentate lacinia mobilis. *Maxilla 1* (Fig. 3F) with nine robust and crenelated setae on inner lobe of which the innermost has a fine comb. A very small vestigial palp is observed. *Maxilla 2* (Fig. 3C) with numerous apical setae, a double row on the inner lobe and a long, finely pinnate seta on its inner margin. *Maxilliped* (Fig. 4A) basal lobe with three blunt teeth on anterior margin, axial margin lined with robust setae armed with setules; palp article 4 reduced to a knob placed between two rows of setae.

**Table 2.** *Cryptorchestia ruffoi* sp. n. Measurements of body length and antennae, showing differences in male and female individuals.

	Total body length	Length of antenna 1	Length of antenna 2
♂	11.60	1.37	4.75
♂ Holotype	15.70	1.64	5.99
♂	14.17	1.91	7.12
♂	10.77	1.29	4.11
♂	10.84	1.36	4.45
♂	10.14	1.21	3.80
**Mean**	**12.20**	**1.46**	**5.04**
♀	11.55	1.20	3.88
♀	12.42	1.54	4.75
♀	10.68	1.15	3.68
♀	11.00	1.00	3.89
♀	10.11	1.01	3.90
♀	11.98	1.16	4.20
♀	11.75	1.17	4.49
♀	10.38	1.00	4.00
♀	11.58	0.90	4.11
♀	10.95	1.04	3.68
**Mean**	**11.24**	**1.12**	**4.06**

Coxae. Coxal plate 1 with numerous robust setae on distal margin. Coxal plates 2–4 wider than deep, plate 5 elongated, bilobate, plates 6 and 7 smaller.

**Figure 5.**
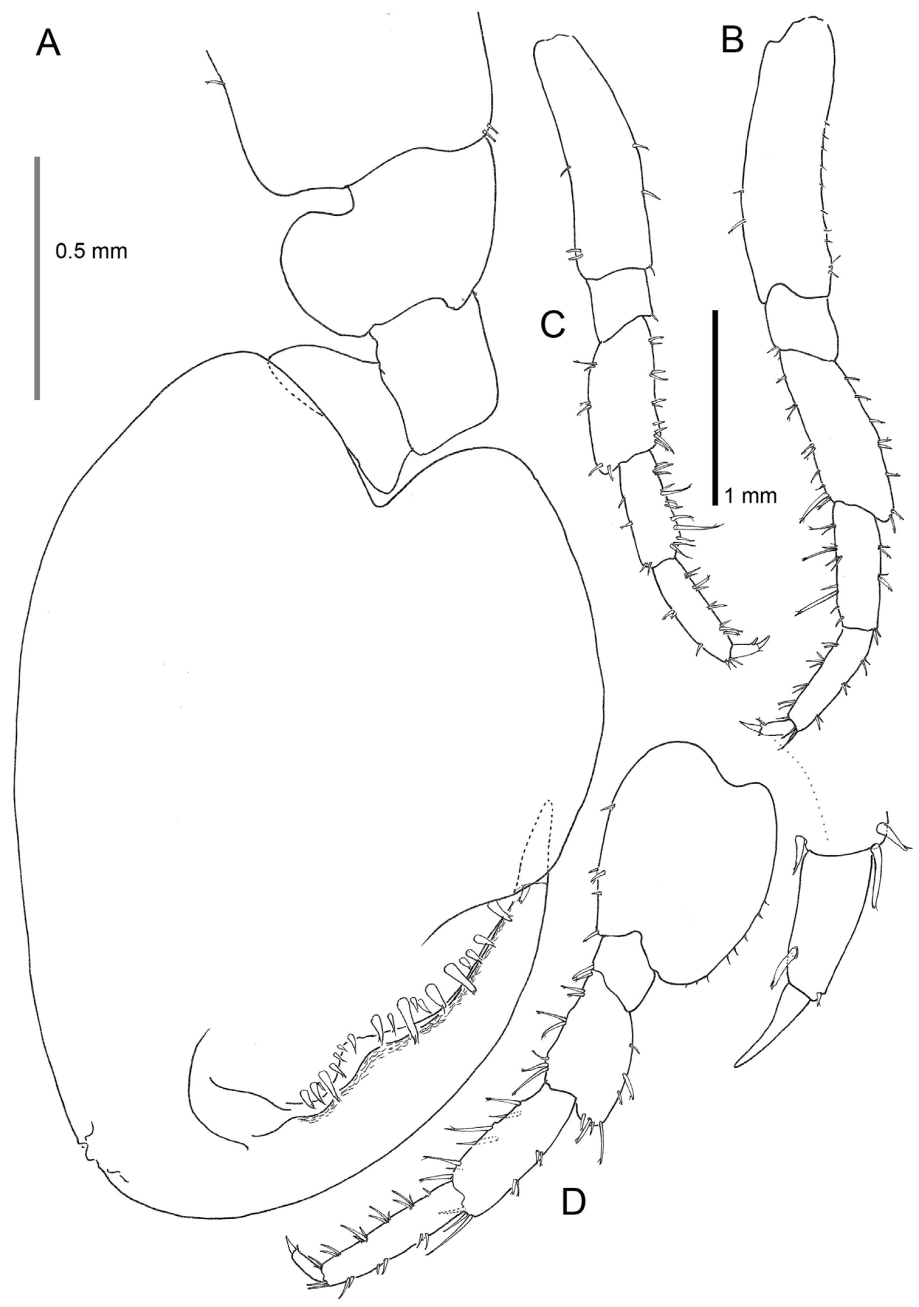
*Cryptorchestia ruffoi* sp. n., paratype male 10.4 mm (RMNH) **A** gnathopod 2 **B** pereopod 4 **C** pereopod 3 **D** pereopod 5.

**Figure 6.**
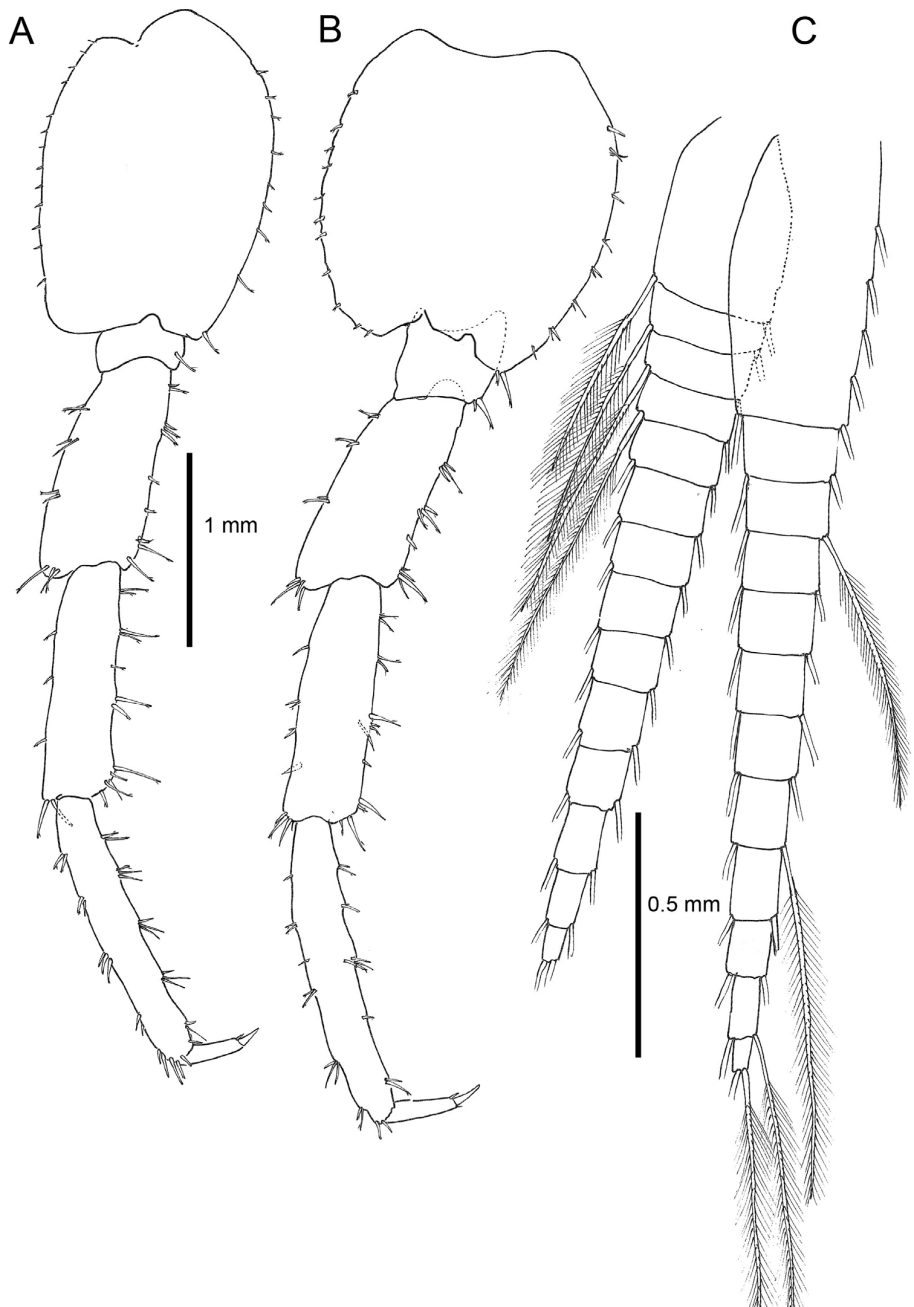
*Cryptorchestia ruffoi* sp. n., paratype male 10.4 mm (RMNH) **A** peropod 6 **B** pereopod 7 **C** pleopod 1.

**Pereon**. *Gnathopod 1* male (Fig. 4C) sexually dimorphic, subchelate; basis with anterior margin lined with six regularly spaced setae, posterior margin with four groups of setae; merus with small partly transparent lobe on posterior margin; carpus with five long setae, rugose at the tips, and placed at the posterior margin; propodus with transverse palm, and with a transparent lobe covering almost the entire palmar margin, and seven short setae lining the palmar margin; dactylus short, slightly longer than anterolateral margin of the propodus. *Gnathopod 2* (Fig. 5A), subchelate; propodus oviform, stout with a rounded protuberance near dactylus insertion, palmar margin with large sinus in the anterodistal part; dactylus somewhat longer than palm. *Pereopods 3–4* (Figs 5B, C) similar; merus of pereopod 3 shorter than that of pereopod 4; dactylus in pereopod 4 with straight inner margin. *Pereopod 5* (Fig. 5D) basis with posterodistal lobe not very wide; propodus with five groups of robust setae on anterior margin. *Pereopods 3–7* cuspidactylate. *Pereopod 6* (Fig. 6A) shorter than pereopod 7; basis elongate; propodus slightly longer than carpus, anterior margin with five groups of long robust setae. *Pereopod 7* (Fig. 6B) basis wide with distinct, rounded posterodistal lobe; merus and carpus not enlarged; propodus longer than carpus.

**Figure 7.**
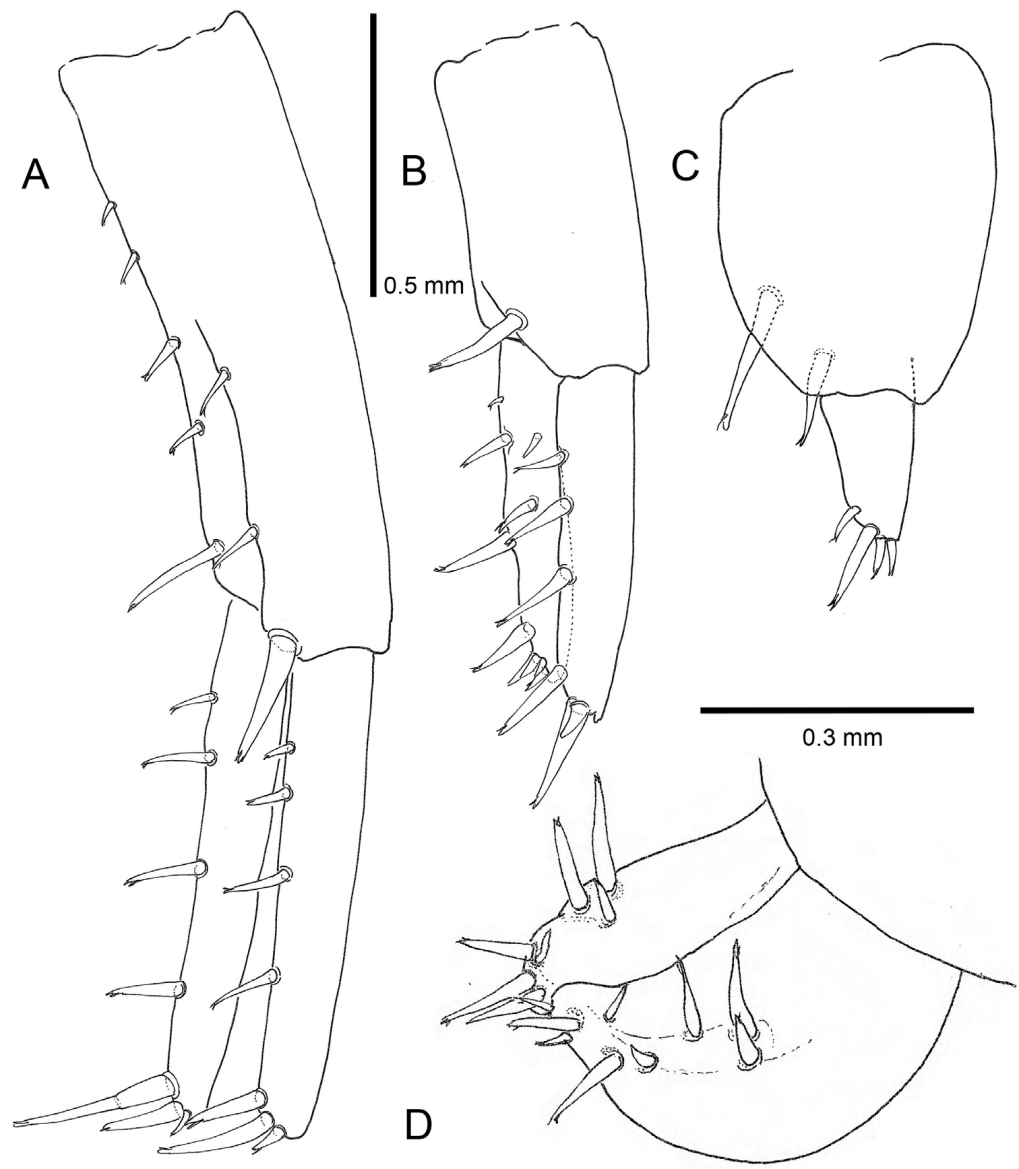
*Cryptorchestia ruffoi* sp. n., paratype male 10.4 mm (RMNH) **A** uropod 1 **B** uropod 2 **C** uropod 3 **D** telson.

**Pleon**. *Epimeral plate 2* with a small posteroventral tooth and almost smooth posterior margin. *Pleopods 1–3* (Fig. 6C) well-developed, biramous, peduncle longer than rami; rami with slender setae; inner ramus slightly shorter than outer. *Uropod 1* (Fig. 7A) with five axial and two medial setae on peduncle, one robust distolateral seta present; outer ramus subequal in length to inner ramus, both with four marginal setae and three apical setae (of which two robust and one small). Uropod 2 (Fig. 7B) peduncle with one robust distolateral seta, inner ramus subequal in length to outer, both with four lateral setae but inner one with two more setae not standing in line. Outer ramus with one strong apical seta and one smaller one, inner ramus with four apical setae. Uropod 3 (Fig. 7C) peduncle with two robust distolateral setae, ramus with four apical setae. Telson (Fig. 7D) longer than broad, dorsal midline entirely cleft, eight marginal and distal robust setae per lobe.

**Figure 8.**
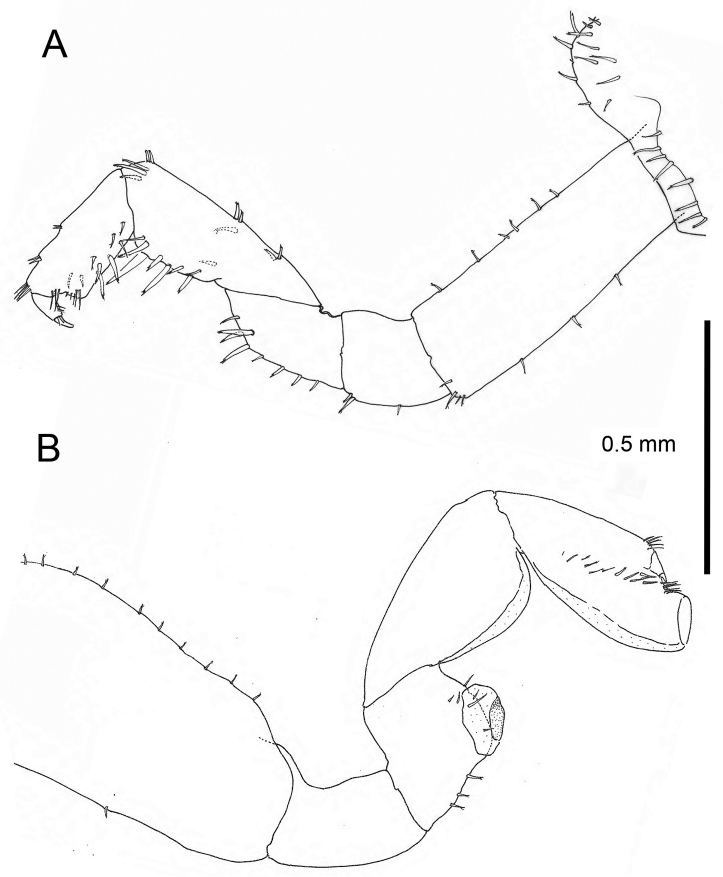
*Cryptorchestia ruffoi* sp. n., paratype female 10.8 mm (RMNH) **A** gnathopod 1 **B** gnathopod 2.

**Female.** Based on adult females with an average length of 11,24 mm (Table 2). *Antenna 1* short, 1.1 mm length in average, flagellum with four articles. *Antenna 2* long, 4 mm in average, flagellum with 15–16 articles. *Gnathopod 1* (Fig. 8A) subchelate; coxal plate lower margin with numerous irregularly placed prominent setae; basis with several short setae, a regular row of three short robust setae on the posterior margin, a less regular row of six setae on the anterior margin; merus with two longer robust setae in a marginal row of 6 smaller ones; carpus with one very robust and long seta between several smaller on lower margin; propodus with three robust setae on lower margin and three bush-like groups on the palmar margin; dactylus slightly longer than palm. *Gnathopod 2* (Fig. 8B), coxa curved, lower margin lined with small short setae; basis with at least 13 short strong setae on anterior margin, and only one in the middle of the posterior margin; ischium without setae; merus with conspicuous bulbous lobe, flattened or incurved at the end, three setae between lobe and proximal margin, some setae present inside lobe; carpus with lobe covering the entire lower margin; propodus with long lobe extending past the palmar margin towards the apex, lobe flattened at fore end; dactylus quite small, shorter than palm. *Oostegites* longer than wide; setae with simple straight tips.

##### Remarks.

**Figure 9.**
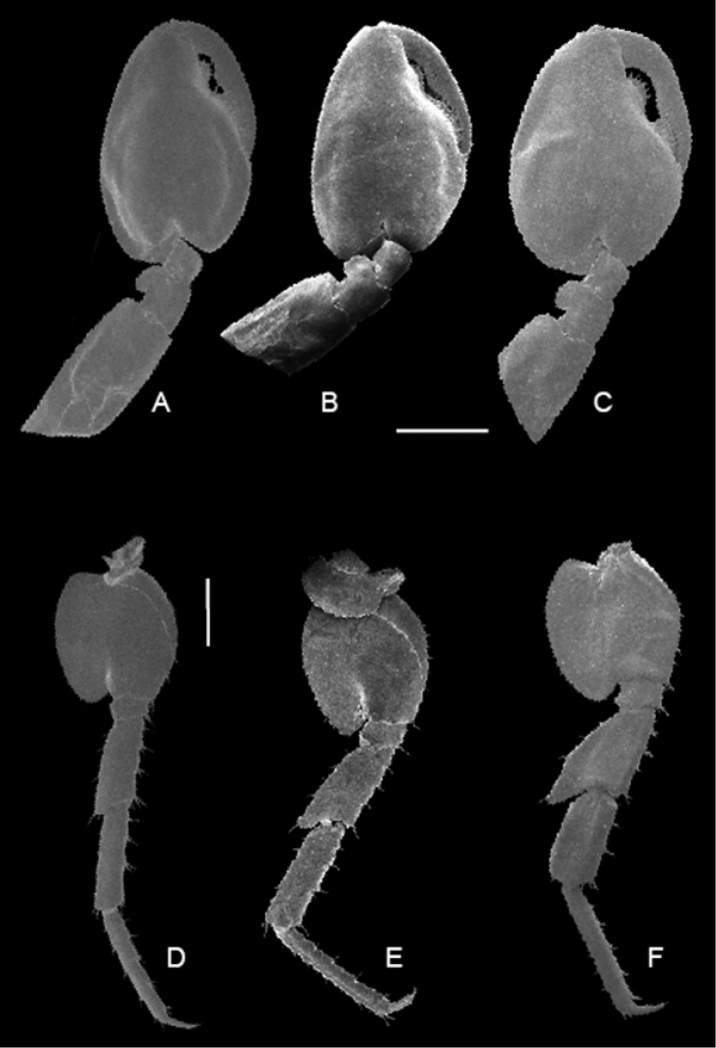
**A** *Cryptorchestia ruffoi* sp. n., gnathopod 2 male, 11.1 mm total body length **B** *Cryptorchestia garbinii* from Lake Garda, gnathopod 2 male, 18 mm total body length **C** *Cryptorchestia cavimana* from Cyprus, gnathopod 2 male, 14. 8 mm total body length, scale bar 1 mm. **D** *Cryptorchestia ruffoi* sp. n. pereopod 7 male **E** *Cryptorchestia garbinii* from Lake Garda, pereopod 7 male **F** *Cryptorchestia cavimana* from Cyprus, gnathopod 2 male, scale bar 1 mm.

The specimens from Rhodes differ in three main characters in comparison to *Cryptorchestia cavimana* (Cyprus), *Cryptorchestia kosswigi* (Ruffo, 1949) (Turkish coast), and *Cryptorchestia garbinii* (Garda Lake), in that they have the sinusoid palmar margin form in the propodus of gnathopod 2 of the male with the strongest incursion closer to the anterior side (Fig. 9A, B, C). *Cryptorchestia ruffoi* sp. n. differs also from *Cryptorchestia garbinii* and *Cryptorchestia cavimana* in the morphology of pereopod 7 basis, merus and carpus (Fig. 9D, E, F). There is also a vestigial palp present on the outer lobe of maxilla 1. This reduced palp has been observed before in *Cryptorchestia monticola* (Madeira). Another regular difference is the presence of four groups of setae on the posterior margin of pereopod 5 in the female specimens and five groups in the males. Out of ten specimens there were three males with five groups and seven females with four groups. A variable difference, in males and females alike, is that in some antennae the first flagellum segment is fused with the second and then both form a longer unit.

Overall, the morphological differences are subtle and perhaps only have meaning in the combination with a unique genetic signature in its COI and H3 gene fragments.

## Results and discussion

**Figure 10.**
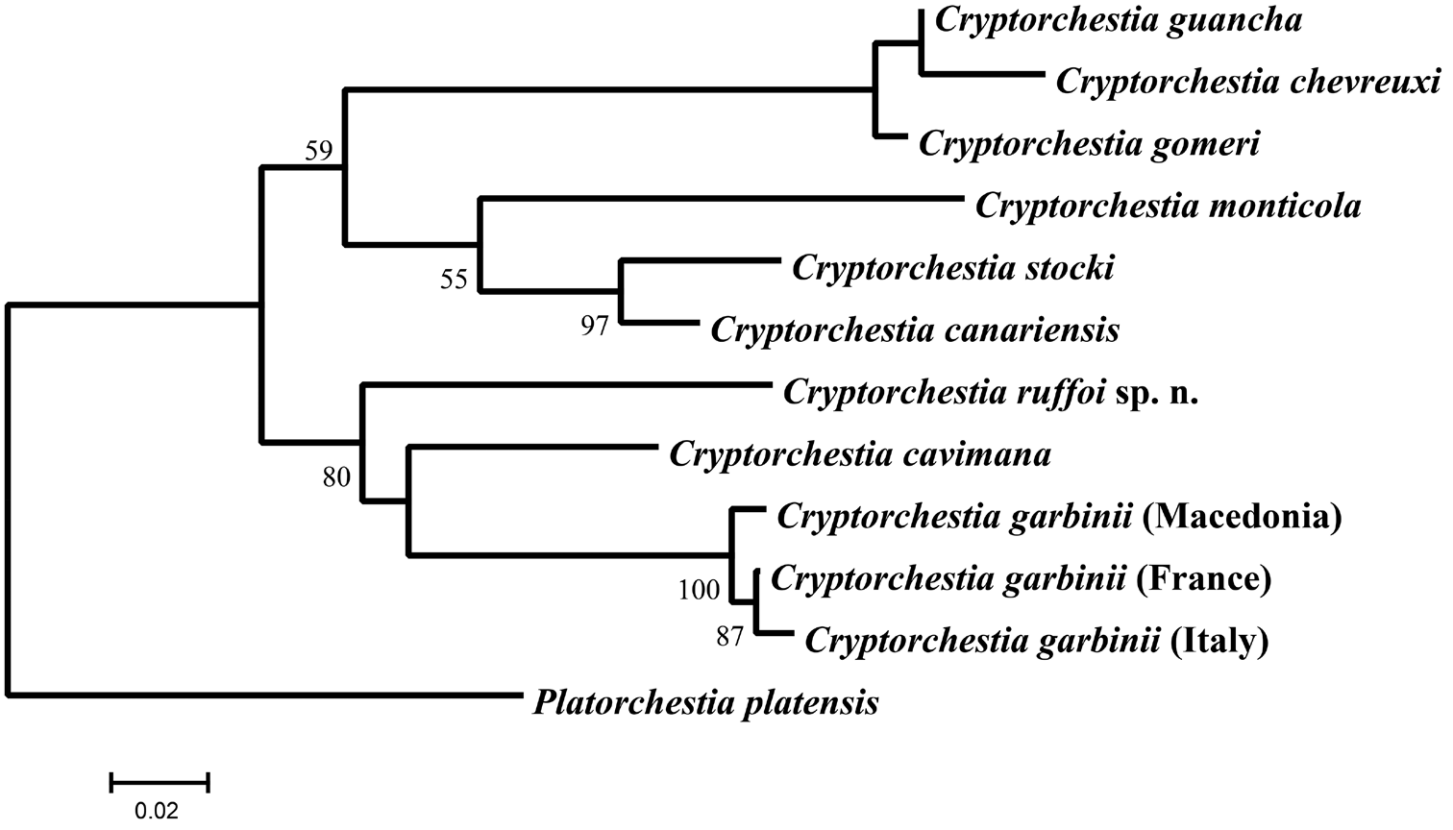
Molecular phylogeny by Maximum Likelihood method obtained in a combined analysis using mitochondrial cytochrome oxidase I (COI) gene region (363 bp), and H3 histone (H3) gene fragment (330 bp) sequences (a total of 693 positions in the final dataset) from *Cryptorchestia ruffoi* sp. n. and other *Cryptorchestia* species reported in Table 1. *Platorchestia platensis* was used in this study as outgroup species. The tree is drawn to scale, with branch lengths measured in the number of substitutions per site. Nodes that have bootstrap values greater than 0.5 are labelled. The GenBank accession numbers of the newly determined sequences from the COI and the histone H3 genes used in this study are reported in Table 1.

The major objective of this molecular study, based on DNA sequences of the mt COI and nuclear histone H3 gene regions, was to estimate the evolutionary relationships of *Cryptorchestia ruffoi* sp. n. in relation to other *Cryptorchestia* species of the Mediterranean and North East Atlantic areas. DNA sequences from the mt region between the COI and COII genes were also analysed; all the species analysed here showed the peculiar rearrangement (data not shown), originally reported in Davolos and Maclean (2005). The phylogenetic scenario based on a Maximum Likelihood method suggested major diversification events, with evolutionary relationships between species generally well supported (Fig. 10). It is possible to recognise a well-supported group including *Cryptorchestia ruffoi* , *Cryptorchestia cavimana*, and *Cryptorchestia garbinii*. This monophyletic group points to a common origin of this *Cryptorchestia* lineage that currently appears to be limited to the East Mediterranean basin. Probably *Cryptorchestia garbinii* has recently colonised Europe, Macedonia (present study) and other regions by a northward expansion (Ruffo et al. 2014). The unique genetic Rhodean *Cryptorchestia* lineage agrees with the species rank of morphological differentiation identified in this study (see taxonomic section for *Cryptorchestia ruffoi* sp. n.). Another outcome of the analyses presented is the presence of a clade that contained the *Cryptorchestia* species (*sensu* Lowry, 2013) of the North East Atlantic area examined in this study: the two closely related species endemic to Gran Canaria: *Cryptorchestia canariensis*, and *Cryptorchestia stocki* (a within-island speciation appears the most-parsimonious hypothesis), *Cryptorchestia monticola* from Madeira, *Cryptorchestia gomeri* from La Gomera, *Cryptorchestia guancha* from Tenerife, and *Cryptorchestia chevreuxi* from Terceira (Fig. 10).

Our results support the proposal by Lowry and Fanini (2013) in that the former *Orchestia cavimana* belongs to a new genus (*Cryptorchestia*). However, the North East Atlantic terrestrial talitrid species, formerly ascribed to *Orchestia*, apparently cannot be included within this new genus (Fig. 10). It is important to bear in mind that *Cryptorchestia ruffoi* sp. n., *Cryptorchestia cavimana*, *Cryptorchestia garbinii*, and *Cryptorchestia kosswigi* from the east Mediterranean regions have a small lobe (probably used in rasping or scrubbing) on the male gnathopod 1 merus as well as on the carpus and propodus (see Ruffo 1949; Ruffo et al. 2014; present study). This character has been proposed to be the main diagnostic difference between *Cryptorchestia* (the type species being *Orchestia cavimana*) and *Orchestia* (in the latter there is a palmate lobe only on male carpus and propodus of gnathopod 1). However, among the northeast Atlantic island terrestrial *Cryptorchestia* species apparently only *Cryptorchestia stocki* (endemic to Gran Canaria as well its closely related species *Cryptorchestia canariensis*) has a small lobe on the merus of gnathopod 1 (Dahl 1950; Stock 1989; Stock and Boxshall 1989; Ruffo 1990; Stock and Abreu 1992). Although previous studies did not reveal that small lobe in *Cryptorchestia canariensis*, a better study of its growth stages could provide further data. The present observations are in line with our DNA sequencing findings that clearly showed a genetic separation of the North-East Atlantic and the Mediterranean *Cryptorchestia* species (Fig. 10). An alternative hypothesis that postulates reduction or loss of the small posterior palmate lobe cannot be disproved, however, our analysis identified similarity in this small structure present on the male gnathopod 1 merus as independently convergent within the east Mediterranean *Cryptorchestia* species and *Cryptorchestia stocki*. Therefore, the occurrence of the small lobe among *Cryptorchestia* species seems to have no clear evolutionary information regarding inclusion in the genus. Overall, the diversification process of *Cryptorchestia* species is of particular interest for future studies. We aim at investigating their evolutionary history by using a larger dataset and multiple calibrations in different parts of a Bayesian inferred phylogeny.

## Supplementary Material

XML Treatment for Cryptorchestia ruffoi
